# Circulating noncoding RNAs: promising biomarkers in liquid biopsy for the diagnosis, prognosis, and therapy of NSCLC

**DOI:** 10.1007/s12672-023-00686-3

**Published:** 2023-08-01

**Authors:** Yilin Li, Jun Ye, Shun Xu, Jiajun Wang

**Affiliations:** Department of Thoracic Surgery, The First Hospital of China Medical University, Shenyang, 110002 China

**Keywords:** NSCLC, Noncoding RNAs, Circulating biomarkers, Liquid biopsy

## Abstract

As the second most common malignant tumor in the world, lung cancer is a great threat to human health. In the past several decades, the role and mechanism of ncRNAs in lung cancer as a class of regulatory RNAs have been studied intensively. In particular, ncRNAs in body fluids have attracted increasing attention as biomarkers for lung cancer diagnosis and prognosis and for the evaluation of lung cancer treatment due to their low invasiveness and accessibility. As emerging tumor biomarkers in lung cancer, circulating ncRNAs are easy to obtain, independent of tissue specimens, and can well reflect the occurrence and progression of tumors due to their correlation with some biological processes in tumors. Circulating ncRNAs have a very high potential to serve as biomarkers and hold promise for the development of ncRNA-based therapeutics. In the current study, there has been extensive evidence that circulating ncRNA has clinical significance and value as a biomarker. In this review, we summarize how ncRNAs are generated and enter the circulation, remaining stable for subsequent detection. The feasibility of circulating ncRNAs as biomarkers in the diagnosis and prognosis of non-small cell lung cancer is also summarized. In the current systematic treatment of non-small cell lung cancer, circulating ncRNAs can also predict drug resistance, adverse reactions, and other events in targeted therapy, chemotherapy, immunotherapy, and radiotherapy and have promising potential to guide the systematic treatment of non-small cell lung cancer.

## Background

Lung cancer is the second most frequently diagnosed cancer and contributes to the highest rates of fatality worldwide. It is estimated that it accounts for approximately 20% of all cancer diagnoses and 27% of cancer deaths in China [1, 2]. Approximately two-thirds of lung cancer deaths can be attributed to tobacco use. Moreover, outdoor and indoor air pollution, which is caused by particulate matter 2.5 μm (PM2.5), coal, and other biomass fuels for heating and cooking, are also major causes of lung cancer [3]. Non-small cell lung cancer (NSCLC) is the main type of lung cancer, accounting for 85% of all patients [4]. Adenocarcinoma, squamous cell carcinoma, and large-cell carcinoma are the three most common types of NSCLC [5]. At present, low-dose computed tomography (LDCT) is an effective screening method for NSCLC. However, it is difficult to screen for NSCLC in some countries with large populations and to distinguish between benign and malignant pulmonary micronodules [6, 7]. Therefore, it is necessary to find a more feasible screening method for the early stages of NSCLC that can also predict prognosis and indicate drug resistance to chemotherapy and tyrosine kinase inhibitors (TKIs). Compared to traditional tissue biopsies, liquid biopsies are relatively easy to implement, sequential available, and minimally invasive, and the use of liquid biopsies in cancer treatment has received a lot of attention over the past decade. Liquid biopsies can be performed on body fluid sample, such as blood, urine, saliva, or cerebrospinal fluid. Biomarkers in the plasma and serum, which are isolated from more readily available peripheral blood, have demonstrated potential application value [8]. For the detection of blood samples, indicators as markers are often circulating tumor cells (CTCS) and CTCS clusters, cell-free DNA (cfDNA)/circulating tumor DNA (ctDNA), extracellular vesicle (ev) and circulating noncoding.

Noncoding RNAs (ncRNAs), which do not encode proteins, play a crucial role in the occurrence and development of NSCLC and remain one of the hotspots in cancer research [9]. They can be categorized as linear RNAs and circular RNAs (circRNAs) according to their structure. Linear RNAs can be categorized as microRNAs (miRNAs) and long noncoding RNAs (lncRNAs) based on their lengths. In general, lncRNAs have a length of more than 200 nucleotides (nt), while miRNAs are only approximately 22 nt in length. Moreover, circRNAs present high stability and can be classified by closed-loop structures that lack 5’ to 3’ polarity and polyadenylated tails.

Studies have found that the expression of circulating ncRNAs are closely related to tumorigenesis, and their abnormal expressions can lead to tumorigenesis, and often change in expression along with tumor progression, which makes ncRNAs potential biomarkers for tumor diagnosis and prognosis [10]. In addition, considering that the expression of ncRNAs is tissue-specific and is often selectively released to extracellular by specific tumor cells through exosomes, specific ncRNA can be used as a specific tumor biomarker to identify tumor sources. Moreover, miRNAs were stably expressed in plasma, serum, urine and saliva. Although the stability of lncRNAs were not as stable as that of miRNAs, it still had a relatively long half-life in serum compared with mRNA with protein coding function [11, 12]. Based on these findings, many studies have selected circulating ncRNA as biomarkers for NSCLC with high specificity and sensitivity. Currently, compared with traditional circulating biomarkers, miRNAs and lncRNAs, circRNAs, as common ncRNAs, have attracted increasing attention and have great potential to be translated into biomarkers for clinical diagnosis and treatment. On the one hand, miRNAs, lncRNAs and circRNAs are stable and are not easily degraded, and they can be obtained by some standard laboratory techniques. In addition, they can be quantified with high sensitivity and specificity through quantitative reverse transcription-polymerase chain reaction (RT‒qPCR), a technique broadly available in clinical laboratories. Furthermore, global profiles can be obtained in a single experiment using RT‒qPCR panels, next-generation sequencing or microarrays [13]. This makes circulating ncRNA as markers of disease diagnosis feasible and convenient for clinical detection.At present, a large number of studies have shown that compared with traditional biomarkers such as carcinoembryonic antigen (CEA) and neuron-specific enolase(NSE), circulating ncRNAs including miR-21, HOTAIR and miR-25 have better sensitivity and specificity for the diagnosis of NSCLC, which also makes them have a very attractive clinical application potential [14–16].

In this review, we summarize the current studies showing the progress of the potential value of miRNAs, lncRNAs, and circRNAs as biomarkers in the diagnosis, prognosis, and evaluation of treatment efficacy for NSCLC.

## The origin of circulating ncRNAs

### Biogenesis of ncRNAs

miRNAs are originally transcribed by RNA polymerase II into primary miRNAs (pri-miRNAs), and then the microprocessor complex, that is, the ribonuclease III enzyme Drosha and the dsRNA-binding protein DGCR8, cleaves pri-miRNAs into precursor miRNAs (pre-miRNAs) before exporting them to the cytoplasm. In the cytoplasm, pre-miRNAs are processed into ds-miRNAs and integrated with the RNA-induced silencing complex (RISC) by Argonaute proteins, leading to mRNA repression or degradation [17].

The biogenesis of lncRNAs occurs in the nucleus, which is similar to protein-coding transcripts. lncRNAs are transcribed by poly II and are capped and polyadenylated at the posttranscriptional level [18, 19] Some lncRNAs are also generated from long primary transcripts with RNA processing pathways, including RNase P cleavage, to generate a mature 3’ end to stabilize the lncRNA [20]. In addition, during the biogenesis of specific lncRNAs, unique subnuclear structures called “paraspeckles” have been identified. Paraspeckles are subnuclear structures generated during the biogenesis of NEAT1 [21], and they may play a role in the regulation of gene expression mediated by lncRNAs, such as the process of the nucleocytoplasmic transport of mRNA [22].

Most circRNAs are transcribed from known protein-coding genes [23] and are circularized by back-splicing (joining the 3′ and 5′ ends together) when the biogenesis of pre-mRNA slows down [24]. Moreover, studies on the mechanism of circularization have been carried out, and formation models such as “lariat-driven circularization” and “intron-pairing driven circularization” have been revealed [25]. “Lariat-driven circularization” forms an exon-containing lariat structure by exon skipping and intron removal to eventually generate an exonic circle. On the other hand, exonic circRNAs (ecircRNAs) and exon–intron circRNAs (EIciRNAs) can be formed by removing introns based on the pairing of complementary motifs in the transcripts [26]. There was also a study that described the formation of circular intronic RNAs (ciRNAs) that occurred due to a failure in debranching, which offers a new perspective on the circularization process in circRNA biogenesis [27].

**Fig. 1:**
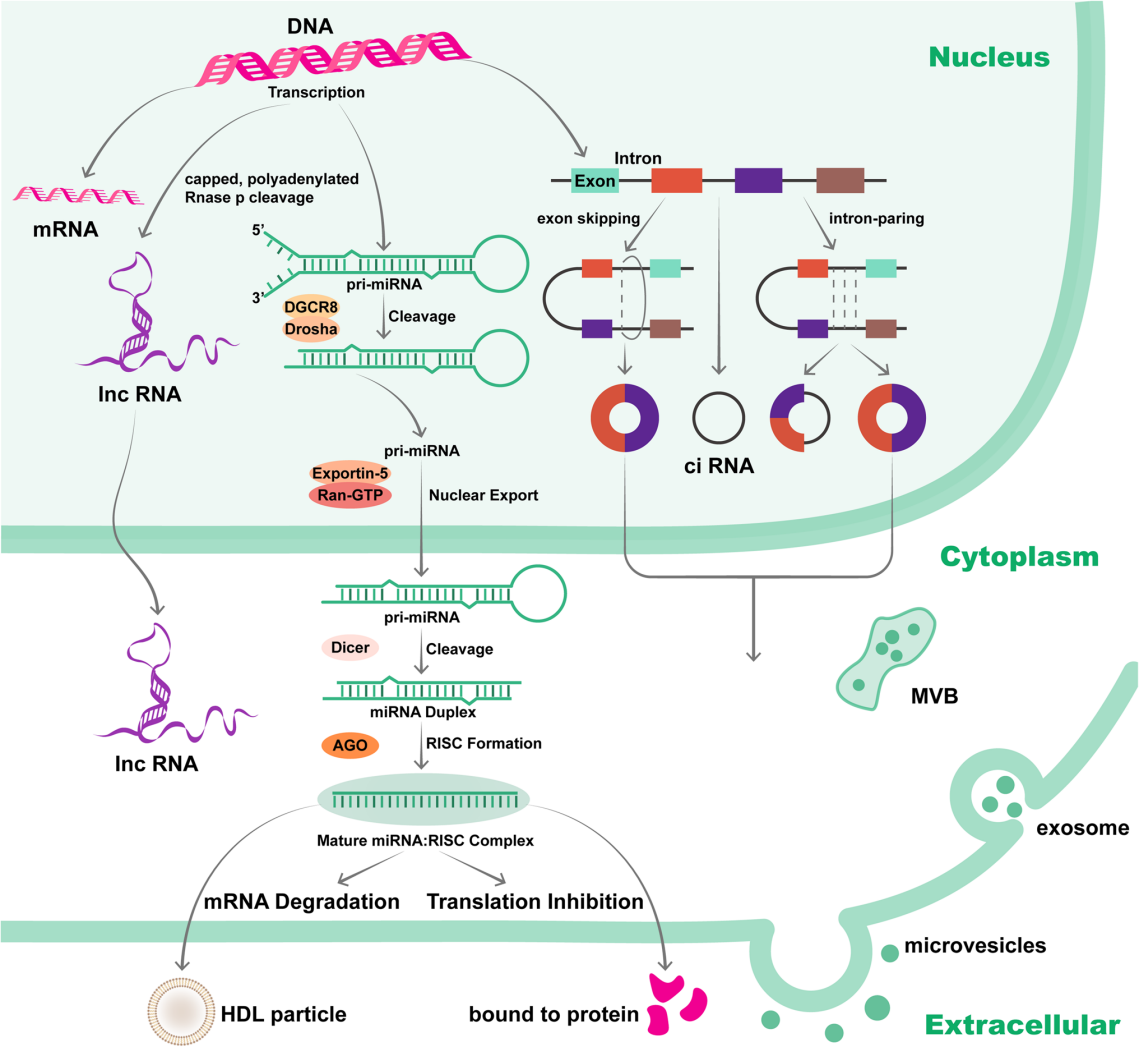
Biogenesis mechanism of ncRNAs

Considering the heterogeneity of different physiological and pathological conditions, the expression of ncRNA is the result of the comprehensive influence of many factors. In the pathological processes associated with tumors, the biogenesis of ncRNAs is regulated by transcription factors, methylation and other processes, so it can reflect the state of tumors at an early stage, which endows ncRNAs with higher sensitivity and specificity as biomarkers. For example, in colorectal cancer, miR-21 is regulated by genetic and epigenetic mechanisms. The promoter of miR-21 is activated or inhibited by histone modification, and miR-21 changes the malignant biological behavior of colorectal cancer by affecting downstream genes [28]. In lung cancer, miR-21 is regarded as a factor that involved in neoplastic processes of transformed human bronchial epithelial cells, and its serum level increases in heavy smokers. Serum miR-21 can effectively distinguish between early NSCLC and benign lung nodules, which may attribute to the mechanism of miR-21 in tumorigenesis [29, 30]. Multiple studies have investigated the potential use of ncRNA expression profiles as biomarkers for cancer diagnosis and prognosis based on tissue specific dysregulation of ncRNA expression in cancer. The levels of circulating ncRNAs are consistent with the trend of abnormal expression in tissues, which may be related to pathways such as extracellular vesicle (EV) release. It was found that miR-451a, miR-194-5p and miR-486-5p were significantly increased in EV in lung adenocarcinoma patients compared with healthy individuals, with an AUC of 0.993[31]. In another study, extracellular vesicle Long RNA (NFKBIA, NDUFB10, SLC7A7, ARPC5, SEPTIN9, HMGN1, H4C2, and Lnc-PLA2G1B-2) were regarded as signature of early NSCLC (diameter < 2 cm), and extracellular vesicle Long RNA can be used as a supplement to LDCT as a noninvasive biomarker for screening early NSCLC [32]. With the progression and change of the disease, the expression level of circulating ncRNA has a sensitive trend of change. Circulating tumor cells (CTCS), as a non-invasive method, also provide an ideal liquid biopsy specimen for screening and diagnosis of early-stage cancers. In patients with epcam-positive metastatic breast cancer, there were differences in the expression levels of miR-21 is almost consistent in the CTCs and plasma, but different between patients and healthy control, suggesting that circulating CTCs-specific miRNA may be a valuable plasma biomarker for distinguishing patients from healthy people in the early stage [33] (Fig. 1).

### How ncRNAs enter the circulatory system and remain stable

Regarding the origin of circulating RNAs, a large number of studies have reported that they do not originate from blood cells, but rather there are other ways in which they are produced [34]. Turchinovich et al*.* indicated that increased expression levels of extracellular miRNAs corresponded to higher cell mortality, which might have been due to the passive release mechanism caused by the apoptosis and necrosis of cancer cells [35]. Apoptotic bodies can also carry ncRNAs in circulation; for example, miR-126 is enriched in apoptotic bodies, and the uptake of apoptotic bodies by recipient cells can cause the transfer of miR-126 [36, 37]. Extracellular vehicles (EVs) play vital roles in the communication between cells and can also package miRNAs, lncRNAs, and circRNAs [38].

RNA-binding proteins (RBPs) are another source of circulating ncRNAs. For example, Vickers et al*.* observed that high-density lipoprotein (HDL) participated in the delivery and transportation of endogenous miRNAs in plasma [39]. In addition, Arroyo et al*.* also observed that Argonaute 2 (Ago2) complexes carry a population of circulating miRNAs in plasma, which also suggests that they provide a mechanism for the stability of plasma miRNAs [40].

Studies have indicated that ncRNAs show relatively high stability in the circulation. In fact, the existence of RNase has been proven in plasma, but endogenous miRNAs can maintain their stability in plasma, while synthetic miRNAs degrade rapidly in plasma [41]. When being incubated at room temperature for 0 and 6 h, undergoing repeated freeze–thaw cycles, and then incubated at − 80 °C, plasma GAS5 remained relatively highly stable [42]. Plasma miRNAs and lncRNA scan remain stable under extreme conditions, which could contribute to the protection of EVs or other molecules, such as RNA–protein complexes [41, 43]. Compared to linear RNA, circRNAs, with their unique circular structure, have better stability in the circulation, and they have a half-life of over 48 h [44], allowing them to serve as potential biomarkers.

## Circulating ncRNAs as diagnosis and prognosis biomarkers of NSCLC

**Fig. 2:**
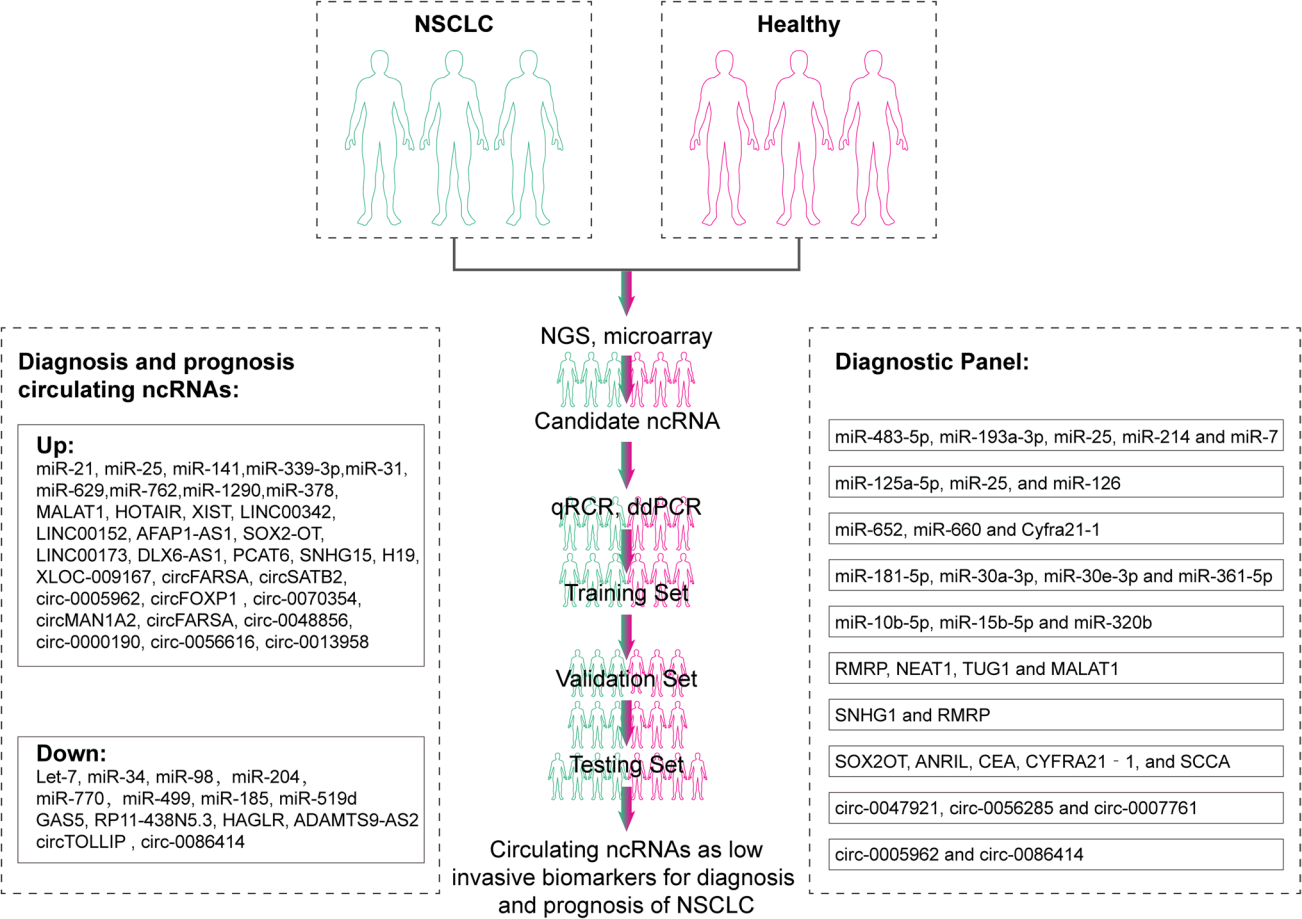
Study method of ncRNAs as diagnostic and prognostic biomarkers miRNAs

Circulating ncRNAs, with their unique features of low invasiveness, high stability, and accessibility, are promising biomarkers for diagnosing NSCLC [45]. In circulation, 90% of plasma cell-free RNAs are ncRNAs [46], and as an important indicator of liquid ncRNA in plasma, they can effectively discriminate NSCLC from healthy donors [46]. Moreover, the detection of a panel of circulating ncRNAs improved the AUC values of diagnoses compared to single ncRNAs and led to more reliable predictions [47]. Meanwhile, studies have also found correlations between the level of circulating ncRNA and the prognosis of NSCLC. Combining the present studies, common circulating ncRNAs and their potential as diagnostic and prognostic biomarkers of NSCLC are summarized as follows (Fig. 2).

### miRNA

#### miR-21

miR-21 contributes to malignant transformation in many cancers, including breast cancer, gastric cancer, and prostate cancer, and regulates many genes in signaling pathways that are involved in cancer progression [48]. In NSCLC, a correlation between the expression level of miR-21 and diagnosis and prognosis has also been determined [49–51]. Compared to other traditional tumor markers, miR-21 obtained the highest AUC of 0.918 [14]. Serum exosomal miR-21 levels could also discriminate NSCLC and benign pulmonary diseases. The miR‐21/let‐7a ratio reached an AUC of 0.8029 in diagnosing NSCLC and 81.33% and 69.57% for sensitivity and specificity, respectively [30]. In addition, miR-21 has the potential to serve as a prognostic biomarker [52]. The high expression of serum miR-21 was associated with lymph node metastasis and an advanced clinical stage of NSCLC, which also indicated poor survival of NSCLC patients [53, 54]. Moreover, the expression profiles of exosomal miRNAs were detected, and exosomal miR-21 was selected as a recurrence marker. Patients in the group with high miR‑21 expression had a significantly worse survival rate [55]. Another study revealed that the expression of miR-21 was also correlated with brain metastasis in NSCLC [56]. As one of the most common ncRNAs, miR-21 has been correlated with many biological processes and could also serve as a biomarker of NSCLC.

#### Let-7

Let-7 was first discovered in C. elegans [57], and the let-7 family has been proven to suppress the proliferation, migration, and invasion of NSCLC by regulating many target genes, such as kRas, ITGB3, and MAP4K3 [58, 59]. As a potential circulating biomarker, Wang et al. confirmed that the plasma levels of let-7 had clinical diagnostic efficiency for NSCLC [60]. By measuring and analyzing the serum level of let-7, the authors found that it had an AUC of 0.771 [61]. In discriminating the pathological progression of NSCLC, circulating let-7 also has potential as a biomarker. According to a study, let-7 was expressed at a relatively lower level in adenocarcinoma in situ (AIS) than in invasive adenocarcinoma, and its expression level could also discriminate the subtypes of AIS [62]. The level of circulating let-7 was associated with prognosis; lower levels of circulating let-7 were associated with the worst NSCLC prognosis [63, 64], and let-7a/b/e/f, which are members of the let-7 family, could specifically predict the prognosis of NSCLC patients [64, 65].

#### miR-25

miR-25 is a member of the miR-106b-25 cluster, which is located within intron 13 of the minichromosome maintenance protein 7 on chromosomes [66]. miR-25 expression is upregulated in NSCLC and can induce biological effects such as oxidative stress to facilitate the progression of NSCLC [67]. Serum miR-25 was also proven to be a biomarker for diagnosing and predicting the outcome of NSCLC [68]. According to a study on 480 patients with NSCLC and 451 healthy controls, miR-25 had an acceptable diagnostic value, with an AUC of 0.85 and specificity of 0.81[69]. Moreover, Zhang et al. found that the combination of plasma CEA and miR-25 could effectively improve the accuracy for distinguishing NSCLC patients from normal controls: the AUC increased from 0.832 to 0.874, which were both better than 0.75 for CEA alone [15]. Circulating miR-25 levels were also an independent prognostic factor that was statistically correlated with lymph node metastasis and the TNM stage of NSCLC [15, 70]. In addition, serum miR-25 was associated with peripheral infiltration and a poor survival rate [71]. Since miR-25 plays a role as a biomarker, it has been repeatedly applied in diagnostic panels of NSCLC.

#### miR-34

The miR-34 family (miR-34a, miR-34b, and miR-34c) is a cluster of tumor suppressors in NSCLC [72] that can directly regulate P53 [73]. Many studies have revealed that its expression is correlated with the diagnosis and prognosis of NSCLC. The high expression levels of plasma miR-34a and miR-34c were correlated with better prognosis [74]. Moreover, miR-34 expression as an independent prognostic factor, along with P53 mutation, might indicate a tendency to relapse in surgically resected NSCLC patients [75]. In addition, the aberrant DNA methylation of miR-34b/c was also correlated with relapse after curative surgery for early-stage NSCLC [76].

#### miR-223

According to a study investigating miR-223 in the plasma of NSCLC, it exhibited relatively high diagnostic value, with an AUC of 0.94 [77]. Specifically, serum miR-223 was a promising biomarker of early-stage NSCLC. D’Antona et al*.* found that miR-223 reached an AUC of 0.753 in a training set of 40 patients with stage I-II NSCLC and 56 controls, and serum miR-223 obtained an AUC of 0.808 in an independent blind set of 35 patients with stage I-II NSCLC and 55 controls [78]. Accordingly, other studies have also supported that circulating miR-223 could be an effective biomarker in distinguishing early-stage NSCLC [79, 80]. Although the current research on miR-223 is limited by the sample size, it still shows very good prospects as a biomarker for NSCLC diagnosis.

#### Circulating miRNAs in panel detection

Regarding the use of a panel for NSCLC diagnosis, many researchers have tried to test the feasibility of a miRNA panel as a diagnostic tool [81–83]. For example, according to a multicenter study on 438 participants from China and the USA, the panel that consisted of miR-483-5p, miR-193a-3p, miR-25, miR-214, and miR-7 was found to have an AUC of 0.976 in diagnosing NSCLC, which was higher than the AUC values for each of the five individual miRNAs, and the diagnostic sensitivity and specificity of the five-miRNA panel for NSCLC diagnosis were 100% and 84%, respectively [84]. Similarly, serum miR-125a-5p, miR-25, and miR-126 were regarded as a diagnostic panel in distinguishing early-stage NSCLC patients from healthy controls, and the panel obtained an AUC of 0.936, which exhibited better efficiency than any of the miRNAs alone [85]. Serum miRNAs were compared with common biomarkers for the diagnosis of NSCLC, and the results indicated that the combination of miR-652, miR-660, and Cyfra21-1 had optimal diagnostic efficiency. In particular, such a model obtained an AUC of 0.941 for the training cohort and an AUC of 0.942 for the test cohort in diagnosing adenocarcinoma [86]. A panel of exosomal miRNAs also showed relatively high efficiency in diagnosing NSCLC. Jin et al*.* detected tumor-derived exosomal miRNAs and identified two diagnostic panels that could discriminate adenocarcinoma, squamous cell carcinoma (SCC), and healthy individuals; the panel consisted of miR-181-5p, miR-30a-3p, miR-30e-3p, and miR-361-5p and could discriminate adenocarcinoma and healthy controls with an AUC of 0.936. In addition, miR-10b-5p, miR-15b-5p, and miR-320b could discriminate SCC and healthy controls with an AUC of 0.911 [87]. Briefly, the detection of a panel of circulating miRNAs could effectively improve the efficiency of diagnosis and outcome prediction, and it is promising that with increased sample sizes, the miRNA panel could be a feasible tool in clinical practice. *Other circulating miRNA panels are shown in* Table 1.

**Table 1:** miRNAs as diagnostic and prognostic biomarkers in NSCLC

miRNA	Expression	Sample Source	Significance of diagnosis and prognosis	The relationship between clinicopathological features	References
miR-141	Up	Serum	Diagnosis: the AUC was 0.856(95% CI: 0.798–0.913); Prognosis: miR-141 ↑ →OS ↓ in adenocarcinoma	Differentiation, lymphatic metastasis, distant metastasis	[88]
miR-339-3p	Up	Serum	Diagnosis: the AUC was 0.616(95% CI: 0.561–0.702), sensitivity: 68.5%, specificity: 55.8%;	NA	[89]
miR-31	Up	Peripheral blood	Diagnosis: the AUC was 0.785(95% CI: 0.486–0.763) sensitivity: 76.9%, specificity: 74.5% Prognosis: miR-31 ↓ → median survival period ↑	NA	[90]
miR-629	Up	Serum	Diagnosis: the AUC was 0.835; Prognosis: miR-629 ↑ → OS, DFS ↓	Lymph node metastasis, differentiation, clinical stage,	[91]
miR-762	Up	Serum	Diagnosis: the AUC was 0.874(sensitivity, 72.97%; specificity, 93.33%); the AUC at the clinical stage I was 0.920(sensitivity, 81.82%; specificity, 93.33%); Prognosis: miR-762 ↑ → OS, RFS ↓	TNM stage, lymph node metastasis, histological grade, gefitinib-resistance	[92]
miR-1290	Up	Serum exosome	Diagnosis: the AUC was 0.937 in LUAD patients (95% CI: 0.890–0.985) Sensitivity: 80.0%, specificity, 96.7% Prognosis: miR-1290 ↑ → PFS ↓	TNM stage, tumor size, lymph node, distant metastasis	[93]
miR-378	Up	Serum exosome	Diagnosis: the AUC was 0.842(sensitivity, 81.5%; specificity, 77.7%); the combination of serum exosomal miR‐378 and CEA yielded the AUC of 0.886(sensitivity, 82.5%; specificity, 84.7%); Prognosis: miR-378 ↑ → OS ↓	Lymph node metastasis, advanced TNM stage	[94]
miR-98	Down	Serum	Diagnosis: the AUC was 0.857 sensitivity:80.3%, specificity: 81.7% Prognosis: miR-98 ↓ → OS ↓	TNM stage, lymph node metastasis	[95]
miR-204	Down	Plasma	Diagnosis**: **the AUC was 0.809 sensitivity: 76%, specificity: 82% Prognosis: miR-204 ↓ → OS, DFS ↓	TNM stage, distant metastasis	[96]
miR-770	Down	Serum	Diagnosis: the AUC was 0.835 (95% CI: 0.751–0.919) sensitivity:68%, specificity:89% Prognosis: miR-770 ↓ → OS ↓	Histological grade, lymphatic metastasis, TNM stage	[97]
miR-499	Down	Serum	Diagnosis: the AUC was 0.906 (95% CI: 0.879–0.929) sensitivity: 73.7%, specificity: 92.7% Prognosis: miR-499 ↓ → OS ↓ miR-499 ↓ → DFS ↓ in stage I-II NSCLC	NA	[98, 99]
miR-185	Down	Serum	Diagnosis: the AUC was 0.790(95% CI: 0.709–0.856) sensitivity: 68.0%, specificity: 78.7% Prognosis: miR-185 ↓ → OS ↓ miR-185 ↓ → recurrent free survival (RFS)↓	Tumor size, lymph node metastasis, TNM stage	[100]
miR-519d	Down	Serum	Diagnosis: the AUC was 0.855 (95% CI: 0.803–0.908) Sensitivity: 98.1%, specificity: 91.8% Prognosis: miR-519d ↓ → OS ↓	Histological grade, lymph node metastases, distant metastases, clinical stage;	[101]

### lncRNAs

#### MALAT1

Metastasis-associated lung adenocarcinoma transcript 1 (MALAT1), as a biomarker in serum/plasma, has relatively high efficiency [102]. It has also shown relatively high diagnostic and prognostic efficiency for NSCLC [103]. Zhang et al. found that serum exosomal MALAT-1 was highly expressed in NSCLC, and the AUC reached 0.703 with a sensitivity of 0.601 and a specificity of 0.809. They also found that the level of serum exo-MALAT-1 was positively associated with TNM stage and lymphatic node metastasis [104]. However, when used as a diagnostic biomarker for specific NSCLC pathological types, MALAT1 may not be very accurate. Schmidt et al*.* detected the expression of MALAT1 and found that high MALAT1 expression was associated with poor prognosis for squamous cell carcinoma [105]. In contrast, a survival cohort analysis depicting the SNP rs3200401 of MALAT1 was associated with advanced lung adenocarcinoma [106]. This controversial result may be due to the different mechanisms of MALAT1 involvement in adenocarcinoma and SCC.

#### GAS5

lncRNA growth arrest-specific transcript 5 (GAS5) is a kind of lncRNA that regulates several cellular functions, including proliferation, apoptosis, invasion, and metastasis [107]. In diagnosing NSCLC, plasma GAS5 yielded relatively high efficiency, with an AUC of 0.832 [42]. According to a study, GAS5 expression was statistically decreased by 29.94% in the plasma of stage I NSCLC patients, and the combined detection of CEA, CA199, and GAS5 performed well in the early diagnosis of NSCLC [108] In addition, circulating GAS5 was confirmed as a biomarker to evaluate and monitor NSCLC surgical resection, with an increasing trend seven days after surgery [42, 108] Tumor‐derived exosomal GAS5 is also a promising biomarker for NSCLC. The AUC was 0.822 when Exo‐GAS5 was used to distinguish patients with early-stage NSCLC, and further analysis implied that Exo‐GAS5 combined with CEA improved the AUC to 0.929. Additionally, Exo-GAS5 was associated with the TNM stage of NSCLC [109].

#### HOTAIR

Homeotic gene transcript antisense RNA (HOTAIR) is a trans-acting intergenic lncRNA that was identified by Howard’s research group[110]. Accumulating evidence suggests that HOTAIR can serve as a biomarker for cancer diagnosis and prognosis [111]. In NSCLC, the plasma level of HOTAIR was measured in 105 patients and 80 healthy controls, and the CEA level was taken as a diagnostic reference. The analysis showed that the combined detection of HOTAIR and CEA (AUC: 0.841) provided a more accurate diagnosis than CEA (AUC: 0.737) or HOTAIR (AUC: 0.791) alone [16]. Another study confirmed that the upregulation of HOTAIR in NSCLC was associated with advanced pathological stage and lymph node metastasis, and patients with high levels of HOTAIR expression had a relatively poorer prognosis [112].

#### lncRNA panel detection

A lncRNA panel showed relatively high feasibility in the diagnosis and prognosis of NSCLC [113]. For example, the stable expression of RMRP, NEAT1, TUG1, and MALAT1 in 528 plasma samples was confirmed. The 4-lncRNA panel obtained an AUC of 0.86 in the training set and an AUC of 0.89 in the verification set in diagnosing NSCLC [114]. SNHG1 and RMRP in plasma were also regarded as a diagnostic panel for NSCLC. The AUC values were 0.9 and 0.8, respectively, for the single detection and the combined detection of these two lncRNAs, showing that they could effectively improve diagnosis accuracy and sensitivity. Furthermore, the fact that the combined detection of the two lncRNAs performed better than that of a single lncRNAs was supported by the low correlation among the levels of the two lncRNAs in a Pearson correlation analysis [115]. In addition, lncRNAs, together with traditional biomarkers in a diagnostic panel, also exhibited relatively high efficiency. Regarding the diagnosis in the specific pathology of NSCLC, the combined detection of the 4-lncRNA (RMRP, NEAT1, TUG1, and MALAT1) panel with traditional tumor markers obtained AUCs of 0.85 for adenocarcinoma and 0.93 for SCC[114] Similarly, Xie et al. reported the expression profile of serum lncRNA in NSCLC and further proposed a diagnostic lncRNA panel consisting of SOX2OT, ANRIL, CEA, CYFRA21‐1, and SCCA, with an AUC of 0.853 in diagnosing NSCLC [116]. Taken together, the results above indicate the potential of the use of a combination of lncRNAs as a biomarker signature for the early detection of NSCLC. Other circulating lncRNA panels are shown in Table 2.

**Table 2:** lncRNAs as diagnostic and prognostic biomarkers in NSCLC

lncRNA	Expression	Sample Source	Significance of diagnosis and prognosis	The relationship between clinicopathological features	References
XIST	Up	Serum	Diagnosis: the AUC was 0.834(95% CI: 0.726–0.935)	NA	[117]
LINC00342	Up	Serum	Diagnosis: the AUC was 0.786 Prognosis**: **LINC00342 ↑ → OS ↓	NA	[118]
LINC00152	Up	Plasma	Diagnosis: the AUC was 0.816(95% CI: 0.757–0.875)	Tumor size, TNM stage, operation effect	[119]
AFAP1-AS1	Up	Serum	Diagnosis: the AUC was 0.759(95% CI:0.692–0.826) sensitivity:69.3%, specificity:88.3%	Distant metastasis, lymph node Metastasis, TNM stage, tumor size	[120, 121]
SOX2-OT	Up	Plasma exosome	Diagnosis: the AUC was 0.815 (95% CI: 0.748–0.882) sensitivity: 76.0%, specificity: 73.17%	Tumor size, TNM stage, lymph node metastasis in patients with SCC	[122]
LINC00173	Up	Serum	Diagnosis: the AUC was 0.809 (95% CI: 0.750–0.868)	Histological type, tumor metastasis	[123]
DLX6-AS1	Up	Serum, exosome	Diagnosis: the AUC was 0.806 sensitivity: 0.775, specificity: 0.859	Advanced stage, positive lymph node metastasis, poor tumor differentiation, surgery effect	[124]
PCAT6	Up	Plasma	Diagnosis: the AUC was 0.9213 (95% CI: 0.8663–0.9763) in LUAD sensitivity:87.67%, specificity:97.44% the AUC was 0.9583 (95% CI: 0.9109–1) in LUSC sensitivity:94.12%, specificity, 100%	NA	[125]
SNHG15	Up	Serum exosome	Diagnosis: the AUC was 0.856(all stage), 0.838(early-stage), 0.870(advanced stage)	Differentiation, lymph node metastasis, TNM stage and surgery effect	[126]
H19	Up	Plasma	Diagnosis**: **the AUC was 0.73 sensitivity, 67.74%; specificity, 63.08%	NA	[127]
XLOC-009167	Up	Whole blood	Diagnosis**: **the AUC was 0.7398 (95% CI: 0.6493–0.8303)	NA	[128]
RP11-438N5.3	Down	Plasma	Diagnosis: the AUC was 0.814(95% CI, 0.743–0.885) Prognosis: RP11-438N5.3 ↑ → OS ↑	TNM stage, distant metastasis	[129]
HAGLR	Down	Plasma exosome	Prognosis: HAGLR ↓ → OS ↑	TNM stage	[130]
ADAMTS9-AS2	Down	Plasma	Diagnosis: the AUC was 0.957 (95% CI: 0.915–0.991) sensitivity: 95%, specificity: 99.1%	TNM stage	[131]

### CircRNAs

CircRNA can serve as a diagnostic and prognostic biomarker in many cancers [132], and they play the same role in NSCLC [133]. For example, Toll-interacting protein (TOLLIP)-derived circRNA (circTOLLIP) can be used as a low-invasive biomarker to distinguish NSCLC from healthy controls. circTOLLIP exists stably in circulation, which allows it to be a circulating biomarker. Peng et al. analyzed the whole blood of 88 patients with NSCLC and 76 healthy controls, and circTOLLIP obtained an AUC of 0.7241 for the discrimination between NSCLC patients and healthy controls. They also confirmed that circTOLLIP achieved better performance than traditional biomarkers, including neuron-specific enolase (NSE), cytokeratin-19-fragment (CYFR21-1), and cancer antigen 72–4 (CA72-4)[134]. On the other hand, circFARSA—that is, a circRNA derived from exons 5–7 of the FARSA gene—was observed in patient plasma. The level of plasma circFARSA was determined in 50 NSCLC cases and 50 healthy controls. Although the relationship between the level of plasma circFARSA and the clinicopathological features of patients has no clear statistical meaning, the AUC was 0.71 in discriminating NSCLC patients and healthy controls [135]. CircRNAs in serum exosomes are also potentially useful tools for low-invasive cancer diagnosis. Zhang et al. found that exosomal circSATB2 could facilitate the progression of NSCLC through the miR-326/FSCN1 pathway, and serum exosomal circSATB2 was higher in metastatic clinical serum samples, which obtained an AUC of 0.797 in distinguishing lymphatic metastatic NSCLC [136]. Moreover, a circular RNA panel also exhibited relatively high efficiency in diagnosing and predicting the outcome of NSCLC. Xian et al. performed an RNA-sequencing analysis of three pairs of NSCLC patients and controls and identified exosomal circ-0047921, circ-0056285, and circ-0007761 as an effective diagnostic panel, with an AUC of 0.926 in the training set and an AUC of 0.919 in the validation set [137]. Similarly, plasma circ-0005962 and circ-0086414 were selected as a diagnostic panel in LUAD with an AUC of 0.81, and further analysis showed that high plasma circ-086414 was related to more EGFR mutations and that circ-005962 displayed potential in the effects of surveillance surgery [138]. Overall, circulating circular RNAs have great potential in serving as diagnostic and prognostic biomarkers, but more research is needed to explore and validate their feasibility based on more samples from the circulatory system of patients (Tables 3, 4).

**Table 3:** circRNAs as diagnostic and prognostic biomarkers in NSCLC

circRNA	Expression	Sample Source	Significance of diagnosis and prognosis	The relationship between clinicopathological features	References
circ-0005962	Up	Plasma	Diagnosis: the AUC was 0.73(sensitivity, 71.90%; specificity, 72.22%) in LUAD	NA	[138]
circFOXP1 (circ-0008234)	Up	Serum	Diagnosis: the AUC was 0.88(95% CI:0.77–0.99)	T stage and lymphatic metastasis	[139]
circ-0070354	Up	Serum	Diagnosis: the AUC was 0.660 (95% CI: 0.589–0.730);	TNM stage, differentiation	[140]
circMAN1A2	Up	Serum	Diagnosis: the AUC was 0.645	NA	[141]
circFARSA	Up	Plasma	Diagnosis: the AUC was 0.71	NA	[135]
circ-0048856	Up	Serum exosome	Diagnosis: the AUC was 0.943 (95% CI: 0.904–0.982) sensitivity: 88.0%, specificity: 80.0%	NA	[142]
circ-0000190	Up	Plasma	Diagnosis: the AUC was 0.95(95% CI: 0.926–0.974) sensitivity: 90.0%, specificity, 90.2%	Tumor size, histological type of adenocarcinoma, stage, distant metastasis,	[143]
circ-0056616	Up	Plasma exosome	NA	Lymph node metastasis in LUAD	[144]
circ-0013958	Up	Plasma	Diagnosis: the AUC was 0.815(95% CI: = 0.727–0.903) sensitivity:75.5%, specificity: 79.6%	TNM stage, lymphatic metastasis	[145]
circ-0086414	Down	Plasma	Diagnosis: the AUC of was 0.78(sensitivity, 77.12%; specificity, 66.67%) in LUAD;	NA	[138]

**Table 4:** ncRNA panel as diagnostic and prognostic biomarkers in NSCLC

miRNA	Sample Source	Role	Reference
miR-191, miR-28-3p, miR-145, miR-328, miR-18a	Serum	Prognosis: associated with the median survival times, and 3-year survival in patients with advanced NSCLC	[146]
miR-20a, miR-21, miR-223, miR-145	Plasma	Diagnosis: the AUC was 0.897 (95% CI, 0.875–0.917) sensitivity: 81.8%, specificity: 90.1%	[79]
miR-942, miR-601	Serum	Diagnosis: the AUC was 0.882; Prognosis: associated with histological grade, lymph node metastases, TNM stage; miR-942 and miR-601 were independent prognostic factors for NSCLC; miR-942 and miR-601 were associated with OS and RFS	[147]
miR-20a, miR-222, miR-221, miR-320, miR-152, miR-145, miR-223, miR-199a-5p, miR-24, miR-25	Serum	Diagnosis: the AUC was 0.966 in training set and the AUC was 0.972 in validation set	[148]
RP11-397D12.4, AC007403.1, and ERICH1-AS1	Plasma	Diagnosis: the AUC was 0.996 in training set and the AUC was 0.942 in validation set	[149]

## Circulating ncRNAs in NSCLC therapy

**Fig. 3:**
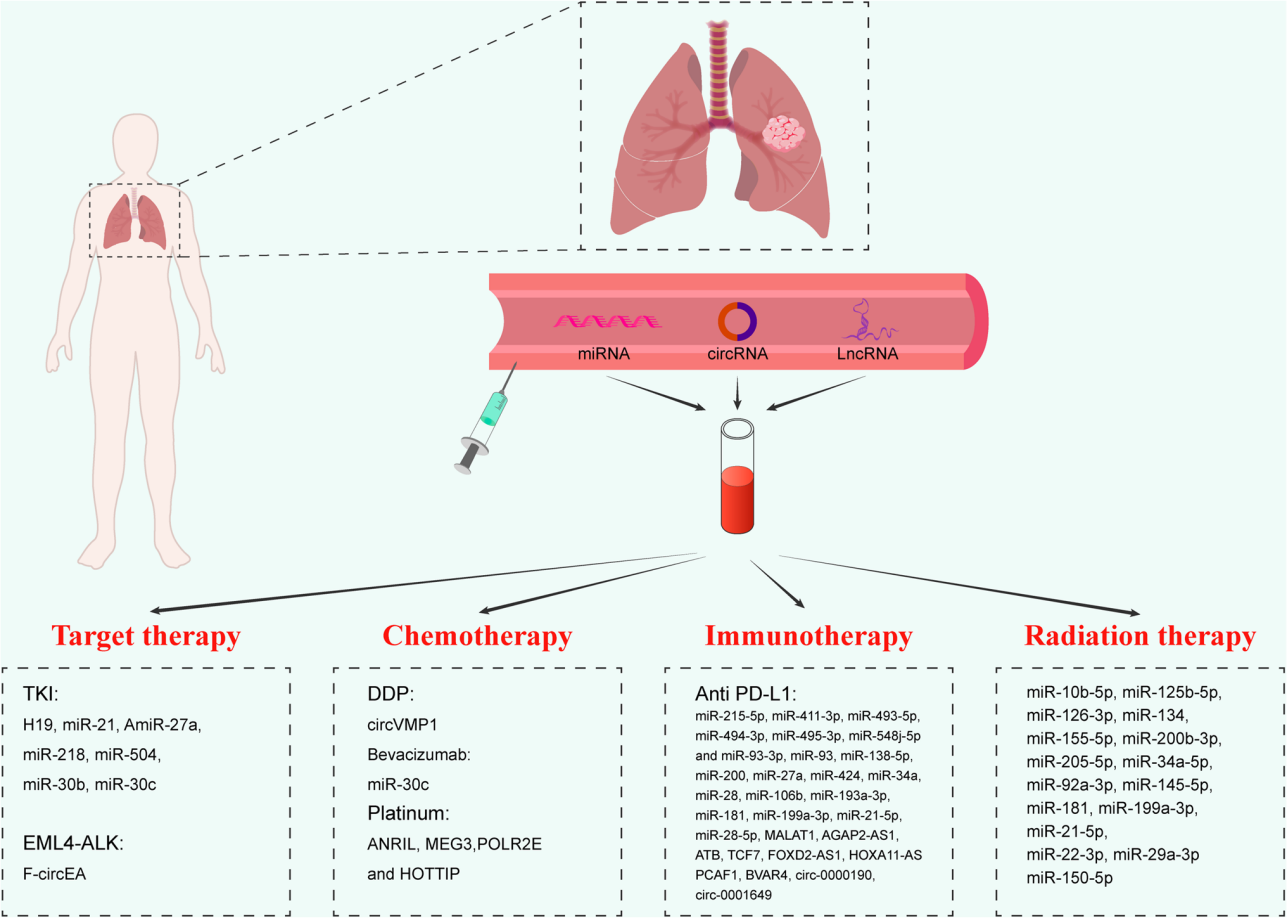
Circulating ncRNAs as biomarkers in the systematic treatment of NSCLC

In the current clinical treatment of NSCLC, radical surgical resection can effectively improve the prognosis of NSCLC. However, not all patients are suitable for surgery [150], and many patients still experience recurrence even after complete resection. For more extensively advanced-stage NSCLC, it is optimal for patients to undergo systemic treatment. For patients with advanced NSCLC, biomarkers are required for the selection of targeted therapy, chemotherapy, or immunotherapy instead of nonselective chemotherapies. In fact, for patients who cannot undergo surgical resection for tissue biopsy, circulating specimens can be utilized to detect biomarkers to distinguish mutations and other statuses for specific therapy. In addition, for patients with drug resistance or drug toxicity in the treatment progress, assessment based on tumor tissues is neither feasible nor capable of being repeated, while the detection of low-invasive circulating biomarkers could potentially identify the molecular mechanism and guide subsequent treatments. In this section, we summarize the studies on the potential of circulating ncRNAs to be used as biomarkers to assist treatment (Fig. 3).

### Circulating ncRNAs as potential biomarkers in targeted therapy for NSCLC

Broadly, NSCLC correlates with gene alterations that have further led to major targeted therapy strategies, such as epidermal growth factor receptor (EGFR), anaplastic lymphoma kinase (ALK), and B-Raf proto-oncogene (BRAF) [151–153]. In addition, molecular biomarkers that monitor the target, predict the response, and guide therapy are still needed. Many ncRNAs are involved in signaling pathways and regulatory networks and can effectively allow for the detection of mutations and factors that mediate resistance. The detection of circulating ncRNAs offers the possibility to dynamically evaluate the efficiency of targeted therapy for NSCLC.

Recent studies have suggested that for advanced NSCLC patients with EGFR mutant tumors, instead of chemotherapy, initial therapy with TKIs may be a better choice. Moreover, some circulating ncRNAs, such as miR-21 and H19, might serve as biomarkers for TKI treatment. miR-21 may induce EGFR-TKI resistance in NSCLC cells by activating the PI3K/AKT pathway through PTEN and PDCD4 inhibition [154], and its circulating expression level has also been correlated with the EGFR-TKI treatment response. To explore the association between miRNA levels and the response to EGFR-TKI treatment, Leonetti et al. dynamically monitored plasma miR-21 levels. The patients were categorized as partial response/complete response (PR/CR) or stable disease/progression of disease (SD/PD) according to their best response, and patients who obtained SD/PD as their best response had a significantly lower baseline value of miR-21 than patients who achieved PR/CR. During the period from two months after treatment to the time of the first radiological evaluation, the miR-21 level was lower in the patients with PR/CR than in those with SD/PD. Of note, the modulation of the plasma miR-21 level indicated the treatment response and could potentially guide therapy [155]. In addition, plasma AmiR-27a and miR-218 together with miR-21 were also verified to have significantly higher expression and potential for indicating primary resistance to EGFR-TKIs in advanced NSCLC patients with EGFR exon 19 deletion mutations [156].

H19 was upregulated in erlotinib-resistant NSCLC cells, and the knockdown of H19 could effectively decrease the resistance of erlotinib in cell viability and IC_50._ Moreover, H19 could be transferred through incorporation into exosomes in erlotinib-resistant NSCLC cells and then induce the resistance of erlotinib by the miR-615-3p/ATG7 axis. Furthermore, serum exosomes were isolated from advanced NSCLC patients receiving erlotinib treatment, and serum exosomal H19 was upregulated in erlotinib-resistant patients. As a potential biomarker for monitoring erlotinib resistance, H19 existed in a stable form in severe situations due to serum exosomes and yielded an AUC of 0.799 in determining the resistance group [157]. Future studies should expand the sample size to further validate the role of circulating H19 in EGFR-TKI treatment. Studies have also indicated that circulating miR-504, miR-30b, miR-30c, and other ncRNAs could serve as biomarkers for EGFR-TKI treatment [158, 159].

It has been reported that miRNA profiles change depending on the mutation target in NSCLC and can effectively discriminate the driver target, such as K-RAS mutation and ALK translocation [160]. As a consequence, ncRNAs are also promising as biomarkers of other mutation targets and improve treatment efficiency. It is common that patients expressing the microtubule-associated protein-like 4 (EML4)–anaplastic lymphoma kinase (ALK) fusion gene may have a poor prognosis when treated with EGFR-targeted inhibitors [161]. Given this occurrence, a new circRNA generated by EML4-ALK fusion (F-circEA) has been proposed. In the clinical validation experiments, F-circEA was detected in the plasma of patients with the EML4-ALK translocation. Despite the lack of large-scale clinical trials, circulating F-circEA offers a new perspective in the recognition of the mechanism of EML4-ALK translocation in NSCLC and is promising as a liquid biopsy biomarker [162].

### Circulating ncRNAs as biomarkers in chemotherapy for NSCLC

Chemotherapy, as a common therapy for cancers, effectively reduces residual tumors and prevents their recurrence [163], but chemoresistance as well as the adverse effects of chemotherapy remain immense challenges for therapy. It has been reported that ncRNAs are involved in the mechanisms of chemotherapy and chemoresistance [164, 165], and circulating ncRNAs are promising biomarkers to monitor resistance and adverse effects. For example, circRNA vacuole membrane protein 1 (circVMP1) was upregulated in cisplatin (DDP)-resistant NSCLC cell lines, and the silencing of circVMP1 could regulate the miR-524-5p-METTL3/SOX2 axis to reduce the IC50 value of DDP, that is, to elevate DDP sensitivity. In the serum of DDP-resistant NSCLC patients, the exosomal circVMP1 level was markedly higher than that in DDP-sensitive patients, and serum exosomal circVMP1 could exist stably under severe conditions to serve as a biomarker in monitoring DDP resistance [166].

Dealing with common adverse effects induced by drug chemotherapy for NSCLC patients, miR-30c has been reported to be an early detection biomarker for predicting the cardiotoxicity caused by chemotherapy. Researchers investigated serum miR-30c levels before chemotherapy, during chemotherapy, and 1 month after chemotherapy, and miR-30c exhibited an AUC of 0.851 in predicting cardiotoxicity in NSCLC patients treated with bevacizumab chemotherapy [167]. The association between lncRNAs and platinum-based chemotherapy gastrointestinal and hematological toxicities was disclosed. In the venous blood of Chinese patients with NSCLC, ANRIL, MEG3, POLR2E, and HOTTIP and their single-nucleotide polymorphisms were analyzed, and the results showed that ANRIL rs1333049 was associated with severe gastrointestinal toxicity, MEG3 rs116907618 was associated with severe gastrointestinal toxicity, and the three-factor interaction model of the POLR2E–rs3787016-HOTTIP–rs3807598-chemotherapy regimen was the best predictive model for hematological toxicity [168].

### Circulating ncRNAs as biomarkers in immunotherapy for NSCLC

Although immune checkpoint inhibitors (ICIs) have broadened the landscape of immunotherapy, primary or secondary resistance is common, and predictive biomarkers for monitoring ICI treatment response may greatly improve treatment efficiency [169]. At present, programmed death-ligand 1 (PD-L1) expression is mainly selected in clinical practice for NSCLC immunotherapy, which is mainly evaluated by tissue biopsy measures, such as immunohistochemistry (IHC) [170], and ICI efficacy is also achieved in PD-L1-negative tumors. Low-invasive circulating biomarkers show promise for guiding immunotherapy in NSCLC patients. Some ncRNAs are involved in immune-related processes in NSCLC and could serve as biomarkers reflecting the response to immunotherapy. For example, the baseline level of plasma exosomal miR-125b-5p showed a significantly increasing trend in progressive disease patients, which could imply the activation of T cells and cytotoxicity to tumor cells in response to immunotherapy and confers resistance [171, 172]. Many studies have explored the feasibility of circulating ncRNAs as biomarkers in immunotherapy for NSCLC. It has been reported that the circulating microRNA signature classifier (MSC) consisting of 21 miRNAs could effectively diagnose NSCLC at an early stage and predict the outcome [173, 174], and the MSC was also a promising biomarker of immunotherapy for NSCLC. The MSC risk levels of 140 patients with NSCLC were prospectively assessed before ICI therapy, and MSCs were statistically associated with the overall response rate (ORR), progression-free survival (PFS), and overall survival (OS). As a supplement to PD-L1, circulating MSCs could effectively identify advanced lung cancer patients with worse ORR, PFS, and OS in immunotherapy regimens [175]. A 7-miRNA signature comprising miR-215-5p, miR-411-3p, miR-493-5p, miR-494-3p, miR-495-3p, miR-548j-5p, and miR-93-3p was also proposed as a predictive biomarker associated with increased OS longer than 6 months after nivolumab treatment [176]. An array analysis was performed on the serum of NSCLC patients receiving nivolumab treatment. The 27-miRNA signature was identified in discriminating responders from nonresponders, and in a validation cohort, a highly expressed 10-miRNA panel (miR-93, miR-138-5p, miR-200, miR-27a, miR-424, miR-34a, miR-28, miR-106b, miR-193a-3p, and miR-181a) was verified to imply better prognosis with nivolumab treatment [177]. EV-associated miRNAs could also be used to monitor immunotherapy response. Shukuya et al. identified 32 miRNAs from plasma and 7 EV-associated miRNAs discriminating responders and nonresponders to immunotherapy, and further analysis indicated that the combined detection of miR-199a-3p, miR-21-5p, and miR-28-5p obtained an AUC of 0.925, predicting patients’ responses to immunotherapy, which was much better than PD-L1 IHC [178]. Of note, a circulating miRNA panel offered a promising measure in monitoring immunotherapy response and prognosis, and several miRNAs have been reported to be relevant to immunotherapy in different studies, which calls for more attention. In addition, in the serum of NSCLC patients with good immunotherapy outcomes, exosomes were isolated, and MALAT1, AGAP2-AS1, ATB, TCF7, FOXD2-AS1, HOXA11-AS, PCAF1, and BVAR4 were proven to be highly expressed, which indicates the potential of serum exosomal lncRNAs as biomarkers for predicting response [177]. Circulating circular RNAs could also serve as a biomarker for immunotherapy. In the analysis of blood samples from patients who received atezolizumab or nivolumab, low/negative expression of PD-L1 and progressive disease were associated with an obvious increase in circRNA levels. In addition, for specific patients with advanced NSCLC receiving immunotherapy, the levels of circ-0000190 and circ-0001649 were more relevant to immunotherapy efficiency than PD-L1 expression in the radiational assessment [143].

### Circulating ncRNAs as biomarkers in radiotherapy for NSCLC

Radiotherapy is a common treatment for locally advanced NSCLC, and the effect is dose dependent. A major challenge is how to increase the radiation dose for cancer cells while reducing damage to surrounding healthy tissue. Many studies have tried to establish a better radiotherapy plan with safety and efficacy [179], and circulating ncRNAs as low-invasive biomarkers have potential in predicting the individualized response of patients to radiotherapy and normal-tissue toxicity [180]. When guiding radiotherapy doses, circulating miRNAs could be used as potential biomarkers to identify individuals who would benefit from a certain radiation dose. Sun et al. investigated a serum miRNA signature predicting the response to high-dose radiation therapy in locally advanced NSCLC. Eleven predictive miRNAs (miR-10b-5p, miR-125b-5p, miR-126-3p, miR-134, miR-155-5p, miR-200b-3p, miR-205-5p, miR-34a-5p, miR-92a-3p, miR-145-5p, and miR-22-3p) were selected and combined with clinical factors to generate a dose‒response score (DRS) for each patient. For patients with a low DRS, high-dose radiation therapy obtained significantly improved OS compared to standard-dose radiation therapy, and the DRS affected the dose response in terms of distant metastasis based on the fact that a higher DRS was correlated with higher dose–hazard ratios [181]. In addition, Dinh et al. performed plasma miRNA profiling at five dose points and identified 10 circulating miRNAs that were correlated with the radiotherapy dose and other dose-dependent indicators. Moreover, in the validation cohort, among the 10 miRNAs, miR-29a-3p and miR-150-5p decreased with increasing radiation doses, and intracellular accumulation and concomitant reduction in exosomes exported from NSCLC and stromal cells could explain the tendency. The two miRNA signatures may reflect a biological tumor response to radiation and serve as potential biomarkers for radiation therapy for NSCLC [182].

When monitoring radiotherapy-induced toxicity, circulating miRNAs could also serve as potential biomarkers to guide treatment. According to a study on predicting radiation‑induced cardiotoxicity in NSCLC, two prognostic models for 14 pretreatment circulating miRNA levels (“c-miRNA”), mean heart dose (MHD) and preexisting cardiac disease (PCD) (“clinical”) were proposed, and both of the models were able to significantly classify patients into high-risk and low-risk groups of developing grade 3 or greater radiation-induced cardiac toxicity. This result implies that circulating miRNAs could obtain the same efficiency as clinical indicators and contribute to patient-specific dose selection and treatment adaptation [183].

## Conclusions and future perspectives

As mentioned above, miRNAs, lncRNAs, and circular RNAs (three common types of ncRNAs) from whole blood, serum, and plasma show high accuracy, sensitivity, and specificity as diagnostic and prognostic biomarkers. In fact, the detection and evaluation of a panel of ncRNAs also revealed their improved efficiency as biomarkers, and there have also been studies combining circulating ncRNAs and traditional protein biomarkers to find an optimal indicator to assist in diagnosis and treatment. Furthermore, the detection of tumor biomarkers from blood samples, as a tool of liquid biopsy, is less invasive and easy to obtain, which could well assist clinical diagnosis and treatment, and the detection of circulating ncRNAs has promising clinical prospects. Despite the rapid dissemination of the research, limitations exist in the current studies on circulating ncRNAs as biomarkers for the diagnosis and treatment of NSCLC and their translation into clinical applications (Figs 1, 2, 3).

First, in the study of ncRNAs as biomarkers, the detection technology for their expression still has limitations, mainly due to preanalytical and analytical factors influencing the data quality [184]. The low concentrations of some ncRNAs in the circulation are not sufficient for the needs of conducting qPCR assays [185], and droplet digital PCR (ddPCR) could be utilized to investigate samples in low abundance. In addition, next-generation sequencing (NGS) has allowed researchers to study genomes at a level never before possible, but this method is still controversial in terms of the selection of sequencing or microarrays for samples in low abundance [186].

Second, clear criteria are needed for sample selection and data analysis. In the current studies, the sample size for testing has usually included dozens to approximately a hundred cases, and studies based on such a sample scale may not be able to fully elucidate the feasibility of circulating ncRNAs as biomarkers for the diagnosis and treatment of NSCLC. Multicenter studies with large-scale sample sizes are needed to further confirm the efficiency of circulating ncRNAs as biomarkers. Larger prospective clinical trials should be conducted to determine whether they reflect results that are more cancer-specific or population specific. For sample selection and quality control, a critical assessment of confounding factors affecting sample handling is needed, which minimizes the variation in circulating ncRNAs detected in different laboratory settings. The levels of ncRNA may differ between the serum and plasma [187], which could be caused by the release of platelets [188] and more studies are needed to establish the inclusion criteria for circulating ncRNA samples. On the other hand, there is also a lack of standards for the selection of sample detection timing. As for the expression level of circulating ncRNA before and after treatment, most studies choose to observe its change trend to determine the effect of treatment. For example, in the dynamic detection of miR-21 after TKI treatment, the baseline level was lower in the patients who received SD/PD within 2 months receiving imaging evaluation. However, many studies lack the detection of expression levels at multiple detection time points and explore its mechanism. Analysis conditions must be standardized across different platforms. Well-regulated, standard operating procedures are essential for the characterization and further validation of circulating ncRNAs. On this basis, large-scale, multicenter, population-intensive validation is more significant in the preclinical stage and provides strong evidence of clinical transformation. In the analysis of detection results, researchers should pay more attention to biomarker tests that can provide greater value for clinical transfomation. In order to obtain higher AUC values, researchers may ignore the fact that blood samples detections are complementary to imaging results: patients with lung nodules indicated by CT often need more specific diagnostic biomarkers. The design and analysis of detection should be closely combined with current imaging technology in clinical diagnosis and treatment to systematically improve the efficiency of clinical diagnosis and treatment.

Third, the selection of reference is a problem. At present, studies choose endogenous reference such as U6 or other exogenous reference. However, considering the limitations of endogenous reference and exogenous reference, for example, there may be differences between endogenous reference and exogenous reference in pathological conditions, such reference cannot fully meet the needs of research. Therefore, a large number of studies are still needed to verify the feasibility and applicability of endogenous and exogenous references. Studies have selected the ncRNA are identically expressed in the NSCLC group and control group as a reference, but simply comparing the differences between tumor patients and non-tumor patients, largely ignore the interference of underlying diseases or metabolic diseases. In fact, blood samples of some people are even obtained from geriatric medicine or other special specialties. For example, some reports suggest that there are differences in ncRNA in hypertensive diabetes [189]. This also leads to the different expression abundance of these references in different populations and pathological specimens, so that the follow-up analysis cannot be repeated and cannot accurately reveal ncRNA biomarkers of universal diagnostic significance. Larger ncRNA profiles of healthy individuals should also be included to identify disease-specific markers and to identify standardized internal parameters to avoid false-positive results.

As we mentioned above, exosomes provide stability for circulating ncRNAs and are correlated with many biological behaviors, and it will be promising to explore the role of exosomes in the investigation of circulating ncRNAs. For example, “Serum Exosomal Long Noncoding RNAs as Potential Biomarkers for Lung Cancer Diagnosis” can be found in the Clinicaltrials.gov database, which reports the sensitivity and specificity of serum exosomal ncRNA as a biomarker for the diagnosis of lung cancer. Because the differential diagnosis of multiple pulmonary nodules requires better strategies, new technologies can be used to explore the role of circulating ncRNA in identifying the origin of pulmonary nodules. An exosome-based imaging tool was developed for the pulmonary metastasis of osteosarcoma by osteosarcoma-derived exosomes as natural nanocarriers. Positron-emitter copper-64 (64 Cu) was utilized to radiolabel OS-derived exosomes, and homologous lung metastatic lesions can be observed by positron emission tomography (PET) [190]. In future studies, exosomes should receive more attention, and there should be a focus on combining corresponding detection techniques for circulating exosome-derived ncRNAs with specific biological behaviors to further improve the function of ncRNAs as diagnostic and therapeutic biomarkers in NSCLC.

## Data Availability

No data were involved.

## Abbreviations

NSCLC: Non-small-cell lung cancer
LDCT: Low-dose computed tomography
TKIs: Tyrosine kinase inhibitors
ncRNAs: Non-coding RNAs
circRNA: Circular RNA
nt: Nucleotides
miRNA: MicroRNA
lncRNA: Long non-coding RNA
mRNA: Messenger RNA
CEA: Carcinoembryonic antigen
NSE: Neuron-specific enolase
CYFR21-1: Cytokeratin-19-fragment
pri-miRNA: Primary microRNA
pre-miRNA: Precursor microRNA
ecircRNA: Exonic circular RNAs
EIciRNAs: Exon–intron circRNAs
ciRNAs: Circular intronic RNAs
RISC: RNA-induced silencing complex
EVs: Extracellular vehicles
RBP: RNA-binding proteins
HDL: High-density lipoprotein
Ago2: Argonaute 2
CTCS: Circulating tumor cells
AIS: Adenocarcinoma in situ
MALAT1: Metastasis associated lung adenocarcinoma transcript 1
GAS5: Growth arrest-specific transcript 5
HOTAIR: Homeotic genes transcript antisense RNA
circTOLLIP: Toll-interacting protein-derived circular RNA
EGFR: Epidermal growth factor receptor
ALK: Anaplastic lymphoma kinase
BRAF: B-Raf proto-oncogene
PR: Partial response
CR: Complete response
SD: Stable disease
PD: Progression of disease
PFS: Progression-free survival
OS: Overall survival
ORR: Overall response rate
DDP: Cisplatin
ICIs: Immune checkpoint inhibitors
MSC: MicroRNA signature classifier
PD-L1: Programmed death-ligand 1
IHC: Immunohistochemistry
DRS: Dose‒response score
MHD: Mean heart dose
PCD: Preexisting cardiac disease
ddPCR: Droplet digital PCR
NGS: Next-generation sequencing
PET: Positron emission tomography
